# Chinese Herbal Medicine for Chemotherapy-Induced Leukopenia: A Systematic Review and Meta-Analysis of High-Quality Randomized Controlled Trials

**DOI:** 10.3389/fphar.2021.573500

**Published:** 2021-05-04

**Authors:** Qing Wang, Hui Ye, Qiu-qin Wang, Wei-tong Li, Bei-bei Yu, Ya-mei Bai, Gui-hua Xu

**Affiliations:** ^1^ School of Nursing, Nanjing University of Chinese Medicine, Nanjing, China; ^2^ Public Teaching Department for Foreign Languages, Nanjing University of Chinese Medicine, Nanjing, China

**Keywords:** traditional Chinese medicine, systematic review, meta-analysis, Chinese herbal medicine, chemotherapy-induced leukopenia

## Abstract

**Aim:** We conducted a systematic review of high-quality randomized controlled trials (RCTs) to assess the efficacy and safety of Chinese herbal medicine (CHM) for the treatment of chemotherapy-induced leukopenia (CIL).

**Methods:** Eight electronic databases were searched from the date of inception to November 4, 2020 for high-quality RCTs that met the requirements of at least four key domains of the Cochrane risk of bias (RoB) tool. RevMan 5.3 was applied for the meta-analysis.

**Results:** Fourteen RCTs involving 1,053 patients were included. The pooled results showed that CHM + chemotherapy exerted greater beneficial effects on white blood cell (WBC), neutrophil (NEU), hemoglobin (Hb), and platelet (PLT) counts in addition to the Karnofsky performance scale (KPS) score, but showed no significant difference on granulocyte colony-stimulating factor (G-CSF) dosage compared with chemotherapy alone. Placebo (PBO) + chemotherapy and CHM + chemotherapy groups showed no significant differences in terms of reduction of the incidence of neutropenia. CHM + chemotherapy was superior to Western medicine (WM) + chemotherapy in improving the WBC count, KPS, infection amount, G-CSF use rate, and incidence of leukopenia. In addition, no severe adverse events were observed in the 14 RCTs.

**Conclusion:** CHM in combination with chemotherapy could effectively improve the clinical symptoms of CIL when compared with chemotherapy alone or Western medicine + chemotherapy, except when comparing with PBO + chemotherapy. While CHMs were generally safe for clinical use and exerted no severe side effects in the 14 RCTs, high-quality RCTs with larger sample sizes are essential to reduce study heterogeneity.

## Introduction

Chemotherapy is widely applied for treatment of multiple cancer types, with one or more anticancer drugs generally used as part of a standardized chemotherapy regimen in the clinic (Johnstone et al., 2002). However, chemotherapy drugs often have poor targeting problems, and combined application of several drugs inevitably results in a series of adverse events (Sarah, 2013), such as bone marrow dysfunction, peripheral neuropathy, chronic pain, sleep disorders, nausea and vomiting, fatigue, and flushes, which not only negatively affect curative effects but also lead to severe patients’ discomfort and poor quality of life (QOL) posttreatment (Torre et al., 2015; Kato et al., 2019; Makary et al., 2019). Bone marrow suppression remains a major toxic effect (Yeshurun et al., 2014), which is characterized by a decrease in three critical cell types: leukocytes, erythrocytes, and platelets. Leukopenia is one of the most prominent effects of bone marrow suppression (Park et al., 2015) and often accompanied by severe infection and bleeding. Long-term usage of cytotoxic drugs is clearly associated with chemotherapy-induced leukopenia (CIL) (Merryman et al., 2012). To increase the white blood cell (WBC) counts within a short time frame for the maintenance of therapeutic efficacy and continue subsequent courses of treatment, granulocyte colony-stimulating factor (G-CSF) is commonly used to treat CIL (Winkler et al., 2016). However, while the effects of G-CSF are rapid, side effects, such as myalgia and fever, are also commonly reported (Yamauchi et al., 2014), making its usage less acceptable in the clinic. Moreover, for patients with severe myelosuppression, repeated treatment is required to maintain the curative effects of chemotherapy. Once medication is stopped, patients are prone to recurrent episodes of illness (Yang et al., 2015). Therefore, treatments that can facilitate effective and stable relief of CIL and promote patients’ QOL by consolidating the clinical value of previous chemotherapeutic regimens and ensuring continuation of therapy are currently a hot topic of research.

Recent studies have shown that traditional Chinese medicine (TCM) aiming to provide personalized treatment plans with multi-targeted and long-lasting effects together with fewer side effects (Zhang et al., 2015) can alleviate the adverse events of conventional chemotherapy (Li et al., 2019). Based on the key treatment concept of syndrome differentiation, TCM has been popularized and widely applied for patients on chemotherapy, including Chinese herbal medicine (CHM), Chinese patent drug, acupuncture, cupping, and other treatments (Li ta al., 2018). Accumulating reports have confirmed beneficial effects of TCM on adverse conditions resulting from bone marrow suppression after chemotherapy. In earlier pharmacological studies, administration of CHM as an adjuvant treatment significantly improved WBC counts in patients with CIL (Liu and Bi, 2015), relieved tumor-related fatigue and dizziness (Han and Jiang, 2018), elevated patients’ QOL (Li et al., 2019), and reduced toxic side effects (Jia et al., 2014). Data from previous meta-analyses clearly suggested that CHM is more effective than conventional oral Western medicine (WM) for the treatment of CIL induced by specific tumors (Li et al., 2015; Zhang et al., 2019), which we find enlightening, but limiting due to different CHM methods and tumor types. Two other systematic reviews and meta-analyses were not limited by the CHM method or tumor type (Li et al., 2016; Niu et al., 2018), but their clinical application and conclusive reliability were unfortunately affected by the low methodological quality of the included literature. Accordingly, we conducted a comprehensive advanced systematic review and meta-analysis of the effects of CHM on CIL, focusing on high-quality RCTs.

## Materials and Methods

Our study was conducted according to the guidelines provided by the Preferred Reporting Item for Systematic Reviews and Meta-Analyses (PRISMA) statement (Moher et al., 2009) and Cochrane Handbook (Higgins et al., 2011).

### Search Strategy

We performed a comprehensive search of 4 English electronic databases (PubMed, Web of Science, Cochrane Library, and Elsevier) from the date of inception to November 4, 2020 and 4 Chinese electronic databases (China National Knowledge Infrastructure, Chinese Biomedical Database, Chinese VIP Information Database, and Wanfang Med Database). The following medical subject heading (MeSH) terms and free text words were used for the search: “Chinese Medicine,” “Chinese Herbal Medicine,” “Chinese patent medicine,” “leukopenia,” “hypoleucocytosis,” “hypolekocytosis,” “neutropenia,” and “bone marrow suppression.” In the Chinese electronic databases, keywords were searched in Chinese characters and Pinyin. There was no limitation on language used.

### Inclusion and Exclusion Criteria

#### Inclusion Criteria

Inclusion criteria were based on the following:

(1) Type of participant: diagnosis of cancer with chemotherapy-induced leukopenia.
(2) Type of study: only high-quality randomized controlled trials (RCTs) related to Chinese herbal medicine in the treatment of CIL, which met the requirements of at least four key domains of the Cochrane risk of bias (RoB) tool, along with trials published in the form of dissertations were selected as eligible studies.
(3) Type of intervention: participants in the intervention groups were treated with CHM in combination with chemotherapeutic drugs. There was no limitation with regard to the form of CHM used (e.g., decoction, capsule, and granule), dosage, or treatment duration. The control groups used chemotherapy alone, chemotherapeutic drugs plus placebo, or chemotherapeutic drugs plus Western medicine, which used to raise leukocytes. All participants were treated *via* oral administration.
(4) Type of outcome measure: primary outcome measures were white blood cell (WBC), neutrophil (NEU), hemoglobin (Hb), and platelet (PLT) counts in addition to the incidence of leukopenia and neutropenia. Secondary outcome measures were the Karnofsky performance scale (KPS) score and improvement, infection amount, granulocyte colony-stimulating factor (G-CSF) dosage and use rate, and adverse events.

#### Exclusion Criteria

Exclusion criteria were as follows:

(1) Patients with leukopenia not caused by chemotherapy.
(2) Duplicate studies, review, animal experiments, and conference abstracts.
(3) Nonoral TCM methods, such as acupuncture, moxibustion, massage, and acupoint injection, in the intervention group or use of CHM drugs in the control group.

### Data Extraction

Two reviewers (QW and HY) independently extracted the relevant data according to the predetermined inclusion and exclusion criteria. The following information was obtained using a standard data extraction form: 1) general information: publication year, language, and first author; 2) characteristics of participants: sample size, age, and gender; 3) intervention information: intervention method, medication dose, and course of treatment; and 4) outcome measures. To resolve any disagreements, the two reviewers discussed the issue or consulted the corresponding author (G-HX).

### Risk of Bias Assessment

The RoB tool was used to assess the methodological quality of included studies (Higgins et al., 2011). Seven aspects were evaluated: random sequence generation, allocation concealment, blinding of participants and personnel, blinding of outcome assessment, incomplete outcome data, selective reporting, and other bias. RCTs that met the requirements of at least 4 of the above parameters were selected for the final analysis.

### Statistical Analysis

RevMan 5.3 was used for statistical analysis. Chi-square test and *I* ^2^ statistic were employed to assess the heterogeneity between intervention and control results. In cases where *I* ^2^ was < 50% or chi-square *p* value was > 0.1, a fixed-effects model was used. Otherwise, a random-effects model was applied. *p* values <0.05 were considered statistically significant. The risk ratio (RR) with 95% confidence interval (CI) was used to calculate the dichotomous data, while the mean difference (MD) or standardized mean difference (SMD) was used to express the continuous data.

### Assessment of Evidence Quality

The overall quality of the evidence for each outcome was assessed by two reviewers (QW and HY) in accordance with the methodology recommended by the Grading of Recommendations, Assessment, Development, and Evaluation (GRADE) criteria (Schunemann et al., 2008). In the system, quality of the evidence varies in four levels: very low quality, low quality, moderate quality, and high quality. Risk of bias, inconsistency, indirectness, imprecision, and other factors (e.g., publication bias) are factors relating to lowering the level of evidence. According to specific criteria such as large *I* ^2^ indicating inconsistency, the level of evidence from RCTs can be downgraded by one or two levels. Summary of findings table for outcomes was performed using GRADEpro GDT.

## Results

### Study Selection

**FIGURE 1:**
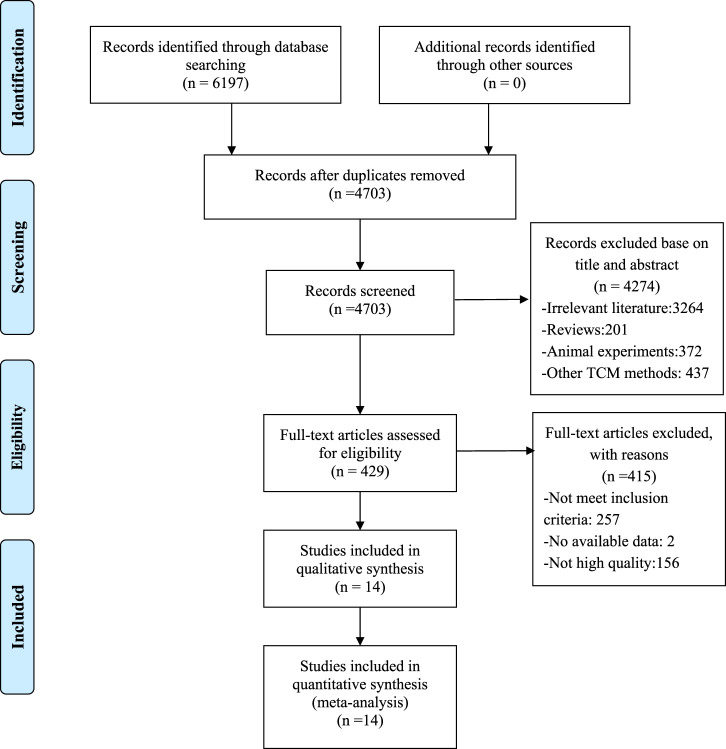
Flowchart of screening process.

A total of 6,197 studies were identified after searching eight databases, from which 1,494 duplicates were removed. Among the remaining 4,703 records, 4,274 were excluded for various reasons after screening according to titles and abstracts. Specifically, 3,264 studies were irrelevant, 437 used other TCM methods, 372 were animal experiments, and 201 were reviews. Comprehensive reading of the full text of the remaining 429 articles resulted in exclusion of 415 studies due to the following reasons: 257 articles did not meet the inclusion criteria, two had no available data, and 156 had low methodological quality. Finally, 14 RCTs with RoB scores ≥4 were included (Mok et al., 2007; Wang, 2011; Liu, 2013; Qian and Li, 2013; Liu and Yao, 2014; Ren and Wu, 2015; Zou, 2015; Wang et al., 2016; Yuan and Zhang, 2016; Zhao and Lu, 2016; Wang and Li, 2017; Li and Liu, 2019; Huang and Zhang, 2020; Li et al., 2020). A flowchart of the screening process is presented in Figure 1.

### Characteristics of Included Studies

A total of 14 studies involving 1,053 patients were included. Only one study was published in English (Mok et al., 2007) and the remaining in Chinese (Wang, 2011; Liu, 2013; Qian and Li, 2013; Liu and Yao, 2014; Ren and Wu, 2015; Zou, 2015; Wang et al., 2016; Yuan and Zhang, 2016; Zhao and Lu, 2016; Wang and Li, 2017; Li and Liu, 2019; Huang and Zhang, 2020; Li et al., 2020). Sample sizes of studies published from 2007 to 2020 ranged from 30 to 120, which included a total of 530 patients in the treatment and 523 in the control groups. Six studies compared CHM + chemotherapy with chemotherapy alone (*n* = 391) (Liu, 2013; Qian and Li, 2013; Liu and Yao, 2014; Zhao and Lu, 2016; Li and Liu, 2019; Huang and Zhang, 2020), and two compared CHM + chemotherapy with placebo (PBO)+ chemotherapy (*n* = 164) (Mok et al., 2007; Yuan and Zhang, 2016). Six studies compared CHM + chemotherapy with WM + chemotherapy (*n* = 498) (Wang, 2011; Ren and Wu, 2015; Zou, 2015; Wang et al., 2016; Wang and Li, 2017; Li et al., 2020). The treatment courses lasted from 14 to 60 days. The preparations used for treatment in the intervention groups of the 14 RCTs were administered orally in the form of decoction (nine comparative analyses), granules (three comparative analyses), capsules, and gel (one comparative analysis separately). The characteristics of the 14 trials are presented in Table 1, and the components are described in Table 2.

**TABLE 1:** Characteristics of the included trials.

First author and publication year	Publication language	Sample size and characteristics (M/F), age (years)		Course of disease	Intervention and dose		Course of treatment (days)	Main outcomes
		Experimental	Control		Intervention	Control		
Liu (2013)	Chinese	28 (20/8)	28 (21/7)	0.1∼7.8 years	Fuzheng Guben decoction, 1 dose, bid + chemotherapy	Chemotherapy	60	WBC count, G-CSF dosage, adverse events
		53.8 ± 3.6	54.2 ± 5.8					
Qian and Li (2013)	Chinese	62 (32/30)	58 (31/27)	NA	Hua Liao Jian du decoction + chemotherapy	Chemotherapy	21	WBC count, Hb count, PLT count, KPS, adverse events
		60.20 ± 7.79	60.40 ± 8.86					
Liu and Yao (2014)	Chinese	38 (20/18)	38 (22/16)	NA	Husui decoction 1 dose, tid + chemotherapy	Chemotherapy	14	WBC count, NEU count, G-CSF dosage, adverse events
		34–64	28–67					
Zhao and Lu (2016)	Chinese	15 (0/15)	15 (0/15)	NA	Fuzheng Shengbai decoction, 1 dose, bid + chemotherapy	Chemotherapy	21	WBC count, NEU count, Hb count, PLT count, KPS, adverse events
		53.07 ± 7.72	48.00 ± 9.80					
Li and Liu (2019)	Chinese	26 (12/14)	26 (11/15)	NA	Jianpi Shengsui gel, 15–20 g, tid + chemotherapy	Chemotherapy	42	WBC count, NEU count, Hb count, PLT count, KPS, adverse events
		53.78 ± 5.95	54.13 ± 6.37					
Huang and Zhang (2020)	Chinese	29 (18/11)	28 (16/12)	NA	Yisui Shengxue capsules 1.8 g, tid + chemotherapy	Chemotherapy	21	WBC count, NEU count, Hb count, PLT count, KPS, adverse events
		62.00 ± 6.35	63.97 ± 6.33					
Mok et al. (2007)	English	55 (5/50)	56 (6/50)	NA	CHM granules, 3–10 g, qd + chemotherapy	Placebo, 3–10 g, qd + chemotherapy	28	Incidence of neutropenia, adverse events
		32–75	39–72					
Yuan and Zhang (2016)	Chinese	27 (15/12)	26 (13/13)	1˜21 month	Qijing Shengbai granules, 12 g, bid + chemotherapy	Placebo 12 g, bid + chemotherapy	42 ± 7	Incidence of neutropenia, adverse events
		59.37 ± 5.869	59.67 ± 6.449					
Ren and Wu (2015)	Chinese	20 (12/8)	20 (13/7)	10˜24 months	Fuzheng Shengbai decoction, 1 dose, bid + chemotherapy	Leukogen tablets 20 mg, tid + chemotherapy	21	WBC count, adverse events
		58.19 ± 8.67	55.5 ± 5.6					
Wang and Li (2017)	Chinese	45 (23/22)	45 (24/21)	NA	Modified Liu Wei Di Huang decoction 1 dose, tid + chemotherapy	Leukogen tablets 20 mg, tid + chemotherapy	21	WBC count, KPS improvement, G-CSF use rate, adverse events
		54.87 ± 8.137	54.73 ± 7.347					
Wang (2011)	Chinese	45 (24/21)	44 (23/21)	NA	Modified Sancai Fengsui decoction, 1 dose, bid + chemotherapy	Leucogen 20 mg and Batyl alcohol 100 mg, tid + chemotherapy	14	WBC count, KPS improvement, infection amount
		35–74	34–75					
Zou (2015)	Chinese	47 (20/27)	48 (21/27)	NA	Sanhuang Sanxian decoction, 1 dose, bid + chemotherapy	Leucogen 20 mg, Batyl alcohol 50 mg, tid + chemotherapy	30	Incidence of leukopenia, infection amount, adverse events
		55.7 ± 16.3	55.5 ± 16.7					
Wang et al. (2016)	Chinese	60 (38/22)	58 (36/22)	NA	Shuanghuang Shengbai granules, 30 g, bid + chemotherapy	Leucogen 40 mg, tid + chemotherapy	14	Incidence of leukopenia, G-CSF use rate, adverse events
		66.17 ± 5.23	66.82 ± 4.96					
Li et al. (2020)	Chinese	33 (12/21)	33 (13/20)	7.64 weeks	Bazhen decoction 400 ml/d + chemotherapy	Leucogen 20 mg, Batyl alcohol 100 mg, tid + chemotherapy	28	WBC count, adverse events
		58.41 ± 8.12	58.35 ± 8.13					

M/F, male/female; NA, not available; WBC, white blood cell; NEU, neutrophil; Hb, hemoglobin; PLT, platelet; KPS, Karnofsky performance scale; G-CSF, granulocyte colony-stimulating factor.

**TABLE 2:** Components of Chinese herbal medicine used in the included trials.

First author and publication year	Chinese herbal medicine	Ingredients of herb prescription		
		Latin name	English name	Chinese name
Liu (2013)	Fuzheng Guben decoction	Processed product of *Glycyrrhiza uralensis* Fisch	Glycyrrhizae Radix et Rhizoma Praeparata Cum Melle	Zhigancao
		Horn processed product of *Cervus nippon* Temminck	Cervi Cornus Colla	Lujiaojiao
		*Rehmannia glutinosa* Libosch	Rehmanniae Radix Praeparata	Shudihuang
		*Psoralea corylifolia* L.	Psoraleae Fructus	Buguzhi
		*Dipsacus asper* Wall. ex Henry	Dipsaci Radix	Xuduan
		*Poria cocos* (Schw.) Wolf	Poria	Fuling
		*Atractylodes macrocephala* Koidz	Atractylodis Macrocephalae Rhizoma	Baizhu
		*Pseudostellaria heterophylla* (Miq.) Pax ex pax et Hoffm	Pseudostellariae Radix	Taizisheng
		*Angelica sinensis* (Oliv.) Diels	Angelicae Sinensis Radix	Danggui
		Processed product of Astragalus membranaceus (Fisch.) Bge.	Astragali Radix Praeparata Cum Melle	Zhihuangqi
Qian and Li (2013)	Hua Liao Jian du decoction	*Panax ginseng* C. A. Mey	Ginseng Radix et Rhizoma Rubra	Hongshen
		*Astragalus* membranaceus (Fisch.) Bge.	Astragali Radix	Huangqi
		*Atractylodes macrocephala* Koidz.	Atractylodis Macrocephalae Rhizoma	Baizhu
		*Angelica sinensis* (Oliv.) Diels	Angelicae Sinensis Radix	Danggui
		*Equus asinus* L.	Asini Corii Colla	Ejiao
		Horn processed product of *Cervus nippon* Temminck	Cervi Cornus Colla	Lujiaojiao
		Processed product of Carapax et Plastrum Testudinis	Testudinis Carapacis et Plastri Colla	Guibanjiao
		Citrμs reticμlata Blanco	Citri Reticulatae Pericarpium	Chenpi
		*Glycyrrhiza* uralensis Fisch	Glycyrrhizae Radix et Rhizoma	Gancao
Liu and Yao (2014)	Husui decoction	Panax ginseng C. A. Mey	Ginseng Radix et Rhizoma	Renshen
		*Astragalus* membranaceus (Fisch.) Bge	Astragali Radix	Huangqi
		*Lycium barbarum* L.	Lycii Fructus	Gouqizi
		Atractylodes macrocephala Koidz	Atractylodis Macrocephalae Rhizoma	Baizhu
		*Rehmannia glutinosa* Libosch	Rehmanniae Radix Praeparata	Shudihuang
		*Angelica sinensis* (Oliv.) Diels	Angelicae Sinensis Radix	Danggui
		*Spatholobus suberectus* Dunn	Spatholobi Caulis	Jixueteng
		*Equus asinus* L.	Asini Corii Colla	Ejiao
		Horn processed product of *Cervus nippon* Temminck	Cervi Cornus Colla	Lujiaojiao
		*Cuscuta australis* R. Br.	Cuscutae Semen	Tusizi
		*Oryza sativa* L.	Oryzae Fructus Germinatus	Daoya
		*Hordeum vulgare* L.	Hordei Fructus Germinatus	Chaomaiya
		*Aucklandia lappa* Decne	Aucklandiae Radix	Muxiang
		*Glycyrrhiza uralensis* Fisch	Glycyrrhizae Radix et Rhizoma	Gancao
Zhao and Lu (2016)	Fuzheng Shengbai decoction	*Curculigo orchioides* Gaertn	Curculiginis Rhizoma	Xianmao
		*Morinda officinalis* How	Morindae Officinalis Radix	Bajitian
		*Ligustrum lucidum* Ait.	Ligustri Lucidi Fructus	Nvzhenzi
		*Eclipta prostrata* L.	Ecliptae Herba	Mohanlian
		*Sanguisorba officinalis* L.	Sanguisorbae Radix	Diyu
		*Pyrrosia lingua* (Bak.) Ching	Pyrrosiae Folium	Shiwei
		*Codonopsis pilosula* (Franch.) Nannf.	Codonopsis Radix	Dangshen
		*Atractylodes macrocephala* Koidz	Atractylodis Macrocephalae Rhizoma	Baizhu
Li and Liu (2019)	Jianpi Shengsui plaster	*Chinemys reevesii* (Gray)	Testudinis Carapax et Plastrum	Guijia
		*Trionyx sinensis* Wiegmann	Trionycis Carapax	Biejia
		Horn of *Cervus nippon* Temminck after extraction	Cervi Cornu Degelatinatum	Lujiaoshuang
		*Codonopsis pilosula* (Franch.) Nannf.	Codonopsis Radix	Dangshen
		*Lycium barbarum* L.	Lycii Fructus	Gouqizi
		*Polygonatum kingianum* Coll. et Hemsl.	Polygonati Rhizoma	Huangjing
		*Ligustrum lucidum* Ait.	Ligustri Lucidi Fructus	Nvzhenzi
		*Eclipta prostrata* L.	Ecliptae Herba	Mohanlian
		Stomach of *Gallus domesticus* Brisson	Galli Gigerii endothelium corneum	Jineijin
		*Hordeum vulgare* L.	Hordei Fructus Germinatus	Maiya
		*Crataegus pinnatifida* Bge	Crataegi Fructus	Shanzha
		*Cyperus rotundus* L.	Cyperi Rhizoma	Xiangfu
		Glucidtemns	Yuanzhen Sugar	Yuanzhentang
		*Equus asinus* L.	Asini corii colla	Ejiao
		*Polygonatum odoratum* (Mill.) Druce	Polygonati Odorati Rhizoma	Yuzhu
		*Ophiopogon japonicus* (L.f) Ker-Gawl	Ophiopogonis Radix	Maidong
Huang and Zhang (2020)	Yisui Shengxue capsule	*Cervus nippon* Temminck	Cervi Cornu Pantotrichum	Lurong
		*Epimedium brevicomu* Maxim	Epimedii Folium	Yinyanghuo
		*Zingiber officinale* Rosc	Zingiberis Rhizoma	Ganjiang
		Panax ginseng C. A. Mey	Ginseng Radix et Rhizoma	Renshen
		*Atractylodes macrocephala* Koidz	Atractylodis Macrocephalae Rhizoma	Baizhu
		*Astragalus* membranaceus (Fisch.) Bge	Astragali Radix	Huangqi
		*Amomum villosum* Lour	Amomi Fructus	Sharen
		Magnetium magnetite	Magnetitum	Cishi
		*Cornus officinalis* Sieb. et Zucc	Corni Fructus	Jiuyurou
		Processed product of polygonum multiflorum Thunb	Polygoni Multiflori Radix Praeparata	Zhiheshouwu
		*Equus asinus* L	Asini corii colla	Ejiao
		*Rheum palmatum* L	Rhei Radix et Rhizoma	Dahuang
		Processed product of *Glycyrrhiza uralensis* Fisch	Glycyrrhizae Radix et Rhizoma Praeparata Cum Melle	Zhigancao
Mok et al. (2007)	CHM granules	A combination of single-itemized herbs from the stocking 225 different types of commonly used herbs		
		(see Appendix for details)		
Yuan and Zhang (2016)	QijingShengbai granules	*Panax quinquefolium* L	Panacis Quinquefolii Radix	Xiyangshen
		*Astragalus* membranaceus (Fisch.) Bge	Astragali Radix	Huangqi
		*Angelica sinensis* (Oliv.) Diels	Angelicae Sinensis Radix	Danggui
		*Eclipta prostrata* L	Ecliptae Herba	Mohanlian
		*Polygonatum kingianum* Coll. et Hemsl	Polygonati Rhizoma	Huangjing
		*Lycium barbarum* L	Lycii Fructus	Gouqizi
		Horn processed product of *Cervus nippon* Temminck	Cervi Cornus Colla	Lujiaojiao
		*Epimedium brevicomu* Maxim	Epimedii Folium	Yinyanghuo
		*Psoralea corylifolia* L	Psoraleae Fructus	Buguzhi
		*Spatholobus suberectus* Dunn	Spatholobi Caulis	Jixueteng
Ren and Wu (2015)	Fuzhengshengbai decoction	*Codonopsis pilosula* (Franch.) Nannf	Codonopsis Radix	Dangshen
		*Astragalus* membranaceus (Fisch.) Bge	Astragali Radix	Huangqi
		*Gastrodia elata* Bl	Gastrodiae Rhizoma	Tianma
		*Angelica sinensis* (Oliv.) Diels	Angelicae Sinensis Radix	Danggui
		*Rehmannia glutinosa* Libosch	Rehmanniae Radix Praeparata	Shudihuang
		*Alisma orientate* (Sam.) Juzep	Alismatis Rhizoma	Zexie
		*Cassia obtusifolia* L	Cassiae Semen	Juemingzi
		*Cuscuta australis* R. Br	Cuscutae Semen	Tusizi
		*Lycium barbarum* L	Lycii Fructus	Gouqizi
		*Eucommia ulmoides* Oliv	Eucommiae Cortex	Duzhong
		*Atractylodes macrocephala* Koidz	Atractylodis Macrocephalae Rhizoma	Baizhu
		*Paeonia lactiflora* Pall	Paeoniae Radix Alba	Baishao
		*Glycyrrhiza uralensis* Fisch	Glycyrrhizae Radix et Rhizoma	Gancao
Wang and Li (2017)	Modified Liu wei di Huang decoction	*Rehmannia glutinosa* Libosch	Rehmanniae Radix Praeparata	Shudihuang
		*Cornus officinalis* Sieb. et Zucc	Corni Fructus	Shanzhuyu
		*Dioscorea opposita* Thunb	Dioscoreae Rhizoma	Shanyao
		*Epimedium brevicomu* Maxim	Epimedii Folium	Yinyanghuo
		*Achyranthes bidentata* Bl	Achyranthis Bidentatae Radix	Niuxi
		*Eucommia ulmoides* Oliv	Eucommiae Cortex	Duzhong
		*Poria cocos* (Schw.) Wolf	Poria	Fuling
		*Alisma orientate* (Sam.) Juzep	Alismatis Rhizoma	Zexie
		*Paeonia suffruticosa* Andr	Moutan Cortex	Danpi
		*Panax ginseng* C. A. Mey	Ginseng Radix et Rhizoma	Renshen
		*Atractylodes macrocephala* Koidz	Atractylodis Macrocephalae Rhizoma	Baizhu
		*Astragalus* membranaceus (Fisch.) Bge	Astragali Radix	Huangqi
		*Crataegus pinnatifida* Bge	Crataegi Fructus	Shanzha
		*Aucklandia lappa* Decne	Aucklandiae Radix	Muxiang
		Massa Medicata Fermentata	Medicated Leaven	Jianqu
Wang (2011)	Modified Sancai Fengsui decoction	*Panax ginseng* C. A. Mey	Ginseng radix et Rhizoma	Renshen
		*Asparagus* cochinchinensis (Lour.) Merr	Asparagi Radix	Tiandong
		*Rehmannia glutinosa* Libosch	Rehmanniae Radix Praeparata	Shudihuang
		*Phellodendron chinense* Schneid	Phellodendri Chinensis Cortex	Huangbo
		*Amomum villosum* Lour	Amomi Fructus	Sharen
		*Astragalus* membranaceus (Fisch.) Bge	Astragali Radix	Huangqi
		*Glehnia littoralis* Fr. Schmidtex Miq	Glehniae Radix	Beishashen
		*Cistanche deserticola* Y. C. Ma	Cistanches Herba	Roucongrong
		*Angelica sinensis* (Oliv.) Diels	Angelicae Sinensis Radix	Danggui
		*Spatholobus suberectus* Dunn	Spatholobi Caulis	Jixueteng
		*Schisandra chinensis* (Turcz.) Baill	Schisandrae Chinensis Fructus	Wuweizi
		*Glycyrrhiza uralensis* Fisch	Glycyrrhizae Radix et Rhizoma	Gancao
Zou (2015)	Sanhuang Sanxian decoction	*Astragalus* membranaceus (Fisch.) Bge	Astragali Radix	Huangqi
		*Polygonatum kingianum* Coll. et Hemsl	Polygonati Rhizoma	Huangjing
		*Scutellaria baicalensis* Georgi	Scutellariae Radix	Huangqin
		*Agrimonia pilosa* Ledeb	Agrimoniae Herba	Xianhecao
		*Epimedium brevicomu* Maxim	Epimedii Folium	Yinyanghuo
		*Curculigo orchioides* Gaertn	Curculiginis Rhizoma	Xianmao
		*Angelica sinensis* (Oliv.) Diels	Angelicae Sinensis Radix	Danggui
		*Paeonia lactiflora* Pall	Paeoniae Radix Alba	Baishao
		*Lycium barbarum* L.	Lycii Fructus	Gouqizi
		*Ligustrum lucidum* Ait	Ligustri Lucidi Fructus	Nvzhenzi
		*Spatholobus suberectus* Dunn	Spatholobi Caulis	Jixueteng
		*Ziziphus jujuba* Mill	Jujubae Fructus	Dazao
Wang et al. (2016)	Shuanghuang Shengbai granules	*Astragalus* membranaceus (Fisch.) Bge	Astragali Radix	Huangqi
		*Astragalus* membranaceus (Fisch.) Bge	Polygonati Rhizoma	Huangjing
		*Drynaria fortune* (Kunze) J. Sm	Drynariae Rhizoma	Gusuibu
		*Ligustrum lucidum* Ait	Ligustri Lucidi Fructus	Nvzhenzi
		*Epimedium brevicomu* Maxim	Epimedii Folium	Yinyanghuo
		*Trichosanthes kirilowii* Maxim	Trichosanthis Radix	Tianhuafen
Li et al. (2020)	Bazhen decoction	*Codonopsis pilosula* (Franch.) Nannf	Codonopsis Radix	Dangshen
		*Astragalus* membranaceus (Fisch.) Bge	Astragali Radix	Huangqi
		Rehmannia glutinosa Libosch	Rehmanniae Radix Praeparata	Shudihuang
		Paeonia lactiflora pall	Paeoniae Radix Alba	Baishao
		*Ligusticum chuanxiong* Hort	Chuanxiong Rhizoma	Chuanxiong
		Poria cocos (Schw.) Wolf	Poria	Fuling
		*Atractylodes macrocephala* Koidz	Atractylodis Macrocephalae Rhizoma	Baizhu
		Angelica sinensis (Oliv.) Diels	Angelicae Sinensis Radix	Danggui
		*Dioscorea opposita* Thunb	Dioscoreae Rhizoma	Shanyao
		*Spatholobus suberectus* Dunn	Spatholobi Caulis	Jixueteng
		*Cuscuta australis* R. Br	Cuscutae Semen	Tusizi
		*Glycyrrhiza* uralensis Fisch	Glycyrrhizae Radix et Rhizoma	Gancao

### Risk of Bias Assessment

Risk of bias (ROB) data are shown in Table 3. All 14 RCTs specified the methods of random sequence generation. Only one study reported the allocation concealment method (Wang and Li, 2017). Two studies blinded both participants and personnel (Mok et al., 2007; Yuan and Zhang, 2016), and one blinded outcome assessment (Mok et al., 2007). As shown in the table, one article scored six points (Mok et al., 2007), two scored five points (Yuan and Zhang, 2016; Wang and Li, 2017), and eleven scored four points (Wang, 2011; Liu, 2013; Qian and Li, 2013; Liu and Yao, 2014; Ren and Wu, 2015; Zou, 2015; Wang et al., 2016; Zhao and Lu, 2016; Li and Liu, 2019; Huang and Zhang, 2020; Li et al., 2020).

**TABLE 3:** Risk of bias assessment of all included studies.

Studies	A	B	C	D	E	F	G	Total
Liu (2013)	+	−	−	−	+	+	+	4+
Qian and Li (2013)	+	−	−	−	+	+	+	4+
Liu and Yao (2014)	+	−	−	−	+	+	+	4+
Zhao and Lu (2016)	+	−	−	−	+	+	+	4+
Li and Liu (2019)	+	−	−	−	+	+	+	4+
Huang and Zhang (2020)	+	−	−	−	+	+	+	4+
Mok et al. (2007)	+	?	+	+	+	+	+	6+
Yuan and Zhang (2016)	+	−	+	?	+	+	+	5+
Ren and Wu (2015)	+	−	−	−	+	+	+	4+
Wang and Li (2017)	+	+	−	−	+	+	+	5+
Wang (2011)	+	−	−	−	+	+	+	4+
Zou (2015)	+	−	−	−	+	+	+	4+
Wang et al. (2016)	+	−	−	−	+	+	+	4+
Li et al. (2020)	+	−	−	−	+	+	+	4+

A, random sequence generation; B, allocation concealment; C, blinding of participants and personnel; D, blinding of outcome assessment; E, incomplete outcome data; F, Selective reporting; G, Other bias. “+” = low risk of bias, “−” = high risk of bias, “?” = unclear risk of bias.

### Assessment of Efficacy

#### Chinese Herbal Medicine Plus Chemotherapy Versus Chemotherapy Alone

**FIGURE 2:**
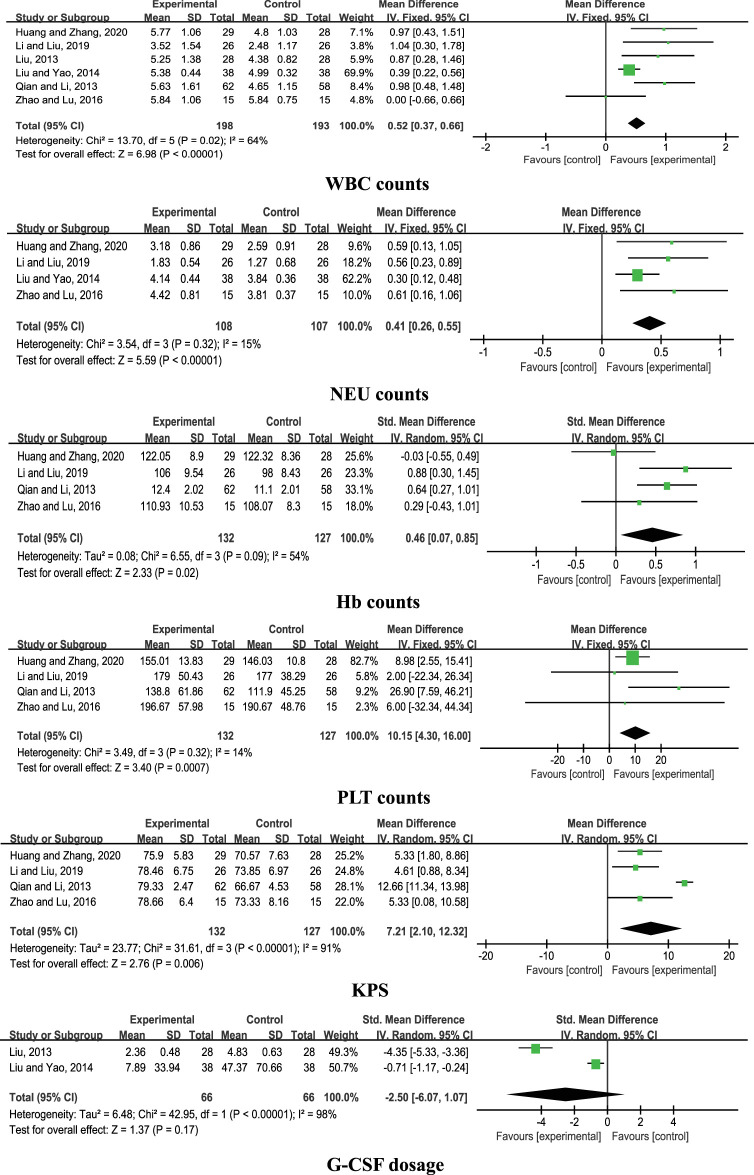
Forest plots of CHM + chemotherpy vs. chemotherapy.

Six comparative studies (Liu, 2013; Qian and Li, 2013; Liu and Yao, 2014; Zhao and Lu, 2016; Li and Liu, 2019; Huang and Zhang, 2020) were included for analysis. The results of the meta-analysis disclosed a significant increase in WBC counts with CHM + chemotherapy, compared with chemotherapy alone (*n* = 391, MD = 0.52, 95% CI: 0.37 to 0.66, *I* ^2^ = 64%, *p* < 0.00001). Data from pooled studies additionally showed that combined treatment with CHM and chemotherapy induced a significant increase in NEU, Hb, and PLT counts and KPS (*p* < 0.00001; *p* = 0.02; *p* = 0.0007; *p* = 0.006), aside from G-CSF dosage (*p* = 0.17; Figure 2).

#### Chinese Herbal Medicine Plus Chemotherapy Versus Placebo Plus Chemotherapy

**FIGURE 3:**
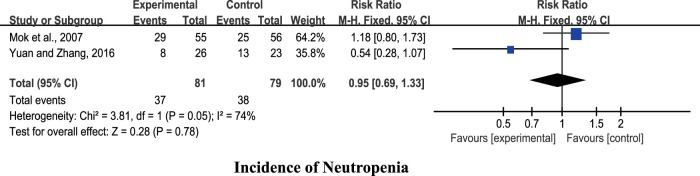
Forest plots of CHM + chemotherapy vs. PBO + chemotherapy.

Two studies (Mok et al., 2007; Yuan and Zhang, 2016) were included for analysis. The pooled results showed no significant differences in reducing the incidence of neutropenia (*n* = 164, RR 0.95, 95% CI: 0.69 to 1.33, *I* ^2^ = 74%, *p* = 0.78; Figure 3).

#### Chinese Herbal Medicine Plus Chemotherapy Versus Western medicine Plus Chemotherapy

**FIGURE 4:**
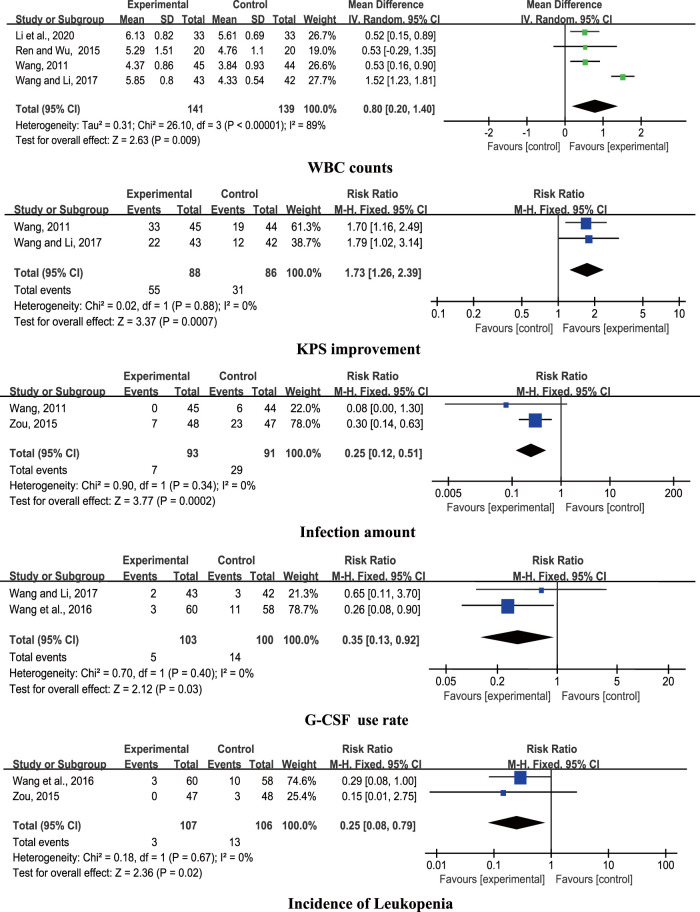
Forest plots of CHM + chemotherapy vs. WM + chemotherapy.

Six studies compared CHM + chemotherapy with WM + chemotherapy (Wang, 2011; Ren and Wu, 2015; Zou, 2015; Wang et al., 2016; Wang and Li, 2017; Li et al., 2020). The pooled results revealed significant differences in WBC counts, KPS improvement, infection amount, G-CSF use rate, and incidence of leukopenia (*p <* 0.05) between the groups (Figure 4).

### Adverse Events

One study (Liu, 2013) reported vomiting and nausea in one case from the experimental group and nine cases from the control group. Both symptoms disappeared after reduction of the treatment dose. Another study (Mok et al., 2007) described occurrence of nausea, vomiting, and anorexia in both the experimental and control groups. In particular, CHM had a significant impact on control of nausea. Two studies (Wang and Li, 2017; Huang and Zhang, 2020) also reported nausea and vomiting in both experimental and control groups, with no significant differences in the two groups. Zou (2015) reported adverse events, such as slight muscle aches, fatigue, low-grade fever, nausea, and vomiting, which were relieved after symptomatic treatment, while Wang et al. (2016) noted one case of mild diarrhea in the intervention group and remission after dosage reduction. No severe adverse events were reported in three studies (Wang, 2011; Ren and Wu, 2015; Yuan and Zhang, 2016). Li et al. (2020) reported rash, dizziness, nausea, and vomiting in the experimental group, which indicated no significant difference compared with the control group. Five other studies (Qian and Li, 2013; Liu and Yao, 2014; Zhao and Lu, 2016; Li and Liu, 2019; Huang and Zhang, 2020) showed no significant differences in liver and kidney functions of patients from both experimental and control groups. The collective results from the 14 RCTs indicate that CHM exerts no severe side effects and is generally safe for human use.

### Assessment of Evidence Quality

Comparing CHM + chemotherapy with chemotherapy alone or PBO + chemotherapy or WM + chemotherapy, the overall quality of evidence according to each outcome measures was moderate or low. The results of GRADE assessments are presented in Table 4.

**TABLE 4:** Assessment of evidence quality.

Outcomes	Anticipated absolute effects		Relative effect (95% CI)	No of Participants (studies)	Quality of the evidence (GRADE)
	Risk with controls	Risk difference with CHM (95% CI)			
**CHM+ chemotherapy vs. chemotherapy**					
WBC counts	—	0.52 MD higher (0.37 to 0.66 higher)	—	391 (6 RCTs)	⊕⊕⊕○ Moderate due to risk of bias
NEU counts	—	0.41 MD higher (0.26 to 0.55 higher)	—	215 (4 RCTs)	⊕⊕⊕○ Moderate due to risk of bias
Hb counts	—	0.46 SMD higher (0.07 to 0.85 higher)	—	259 (4 RCTs)	⊕⊕○○ Low due to risk of bias, inconsistency
PLT counts	—	10.15 MD higher (4.3 to 16.0 higher)	—	259 (4 RCTs)	⊕⊕⊕○ Moderate due to risk of bias
KPS	—	7.21 MD higher (2.1 to 12.32 higher)	—	259 (4 RCTs)	⊕⊕○○ Low due to risk of bias, inconsistency
G-CSF dosage	—	2.5 SMD lower (6.07 lower to 1.07 higher)	—	132 (2 RCTs)	⊕⊕○○ Low due to risk of bias, inconsistency
**CHM+ chemotherapy vs. PBO+ chemotherapy**					
Incidence of neutropenia	—	—	RR 0.95 (0.69 to 1.33)	160 (2 RCTs)	⊕⊕○○ Low due to inconsistency, imprecision
**CHM+ Chemotherapy vs. WM+ Chemotherapy**					
WBC counts	—	0.80 MD higher (0.20 to 1.40 higher)	—	280 (4 RCTs)	⊕⊕○○ Low due to risk of bias, inconsistency
KPS improvement	—	—	RR 1.73 (1.26 to 2.39)	174 (2 RCTs)	⊕⊕⊕○ Moderate due to risk of bias
Infection amount	—	—	RR 0.25 (0.12 to 0.51)	184 (2 RCTs)	⊕⊕⊕○ Moderate due to risk of bias
G-CSF use rate	—	—	RR 0.35 (0.13 to 0.92)	203 (2 RCTs)	⊕⊕⊕○ Moderate due to risk of bias
Incidence of leukopenia	—	—	RR 0.25 (0.08 to 0.79)	213 (2 RCTs)	⊕⊕⊕○ Moderate due to risk of bias

RCT, randomized clinical trial; SMD, standardized mean difference; MD, mean difference; CI: confidence interval. High quality: Further research is very unlikely to change our confidence in the estimate of effect. Moderate quality: Further research is likely to have an important impact on our confidence in the estimate of effect and may change the estimate. Low quality: Further research is very likely to have an important impact on our confidence in the estimate of effect and is likely to change the estimate. Very low quality: We are very uncertain about the estimate.

## Discussion

Cancer remains a severe public health problem with rapidly increasing incidence and mortality rates and is the leading cause of death worldwide (Bray et al., 2018; Siegel et al., 2020). Chemotherapy is currently the main treatment modality for cancer. However, CIL is the most common adverse effect of chemotherapy, which is directly associated with survival rates (Liu et al., 2013; Campos et al., 2015; Wyatt et al., 2015; Varughese et al., 2020). G-CSF is commonly used to relieve side effects and improve the QOL of patients with CIL (Disis, 2005; Ohnaka et al., 2013; Cornes et al., 2018). However, despite rapid effects, the drug is not suitable for long-term use in all patients owing to its high cost and secondary malignancy risk (Lyman et al., 2013; Lyman, et al., 2018). Thus, increasing number of patients have turned to complementary and alternative medicines to control symptoms and improve the QOL (Kuo et al., 2018; Knecht et al., 2020). CHM has received considerable research attention over the past few years and is widely applied following chemotherapy for various cancer types. Compared with G-CSF, the long-lasting effects and affordability of CHM have made it the preferred treatment of choice for many patients in China. Based on continued evaluation of clinical efficacy from meta-analyses together with accumulating experimental and pharmacological insights into their mechanisms of action, CHM drugs could increasingly benefit patients with chemotherapy-induced leukopenia (CIL) worldwide (Jia et al., 2015; Yang et al., 2017; Chen et al., 2018).

Trials included in previous meta-analyses (Li et al., 2016; Niu et al., 2018) had low methodological quality, with no information on blinding and allocation concealment. This updated systematic review included 14 high-quality studies that were divided into three subgroups to reduce heterogeneity, with a total of 1,053 selected patients. Earlier studies by Li et al. (2016) and Niu et al. (2018) had few evaluation indicators, and both showed superiority of CHM in improving the clinical efficacy rate. Niu and coworkers (2018) additionally demonstrated that CHM could enhance the KPS score.

In the current meta-analysis, the methodological quality was adjusted, the latest literature was included, and more specific indicators were evaluated, with the aim of providing a comprehensive and reliable reference for subsequent research. Pooled data on comparative analyses of CHM + chemotherapy and chemotherapy alone revealed that CHM had greater beneficial effects on several indicators, including WBC, NEU, Hb, and PLT counts, as well as KPS, but not G-CSF dosage. The CHM + chemotherapy group was non-inferior to the PBO + chemotherapy group for the incidence of neutropenia, supporting the findings of Liew et al. (2019) and Mok et al. (2007); CHM did not reduce hematologic toxicity associated with chemotherapy. The CHM + chemotherapy group was superior to the WM + chemotherapy group in terms of improvement of the WBC counts, KPS score, infection amount, G-CSF use rate, and incidence of leukopenia. In addition, CHM drugs were generally safe and induced no severe adverse events.

Our study has several limitations that should be taken into consideration. First, the methodological quality of the included studies was generally low. Although we searched both English and Chinese databases, potential selection bias may have been introduced since most of the included records retrieved were Chinese. Second, although all the trials met the requirements of at least four parameters of the Cochrane RoB tool, some methodological restrictions existed in primary studies. Only one study reported the method of allocation concealment that could lead to selection bias. Only two described blinding of participants and personnel, and one described blinding of outcome assessment. Missing blinding may cause detection bias. It is difficult to conduct adequate blinding in CHM RCTs due to different smells and tastes of CHM decoctions. Third, different components, doses, and duration of CHM interventions may have caused potential heterogeneity. Fourth, some indicators appeared less frequently, only in two studies. Finally, the small sample of subgroup and the quality of the evidence lead the conclusion to be more objective and explicit, giving us inspiration to design related clinical intervention.

## Conclusion

CHM in combination with chemotherapy could improve clinical symptoms of CIL when compared with chemotherapy alone or Western medicine + chemotherapy, except when comparing with PBO + chemotherapy. While CHMs were generally safe and exerted no severe side effects in all 14 RCTs, larger sample sizes and high-quality RCTs are required to reduce study heterogeneity.

## Appendix

*Radix Scrophulariae*, *Polyporus*, *Coix Lacrymajobi*, *Semen Plantaginis*, *Herba Lysimachiae*, *Spora Lygodii*, *Herba Artemisiae Scopariae*, *Fructus Kochiae*, *Herba Dianthi*, *Herba Plantaginis*, *Pericarpium Arecae*, *Rhizoma Smilacis Glabrae*, *processed Radix Aconiti*, *Rhizoma Zingiberis*, *Cinnamomun cassia*, *Herba Asari*, *Fructus Evodiae*, *Radix Aconiti Kusnezoffii*, *Citrus Reticulata*, *Fructus Auranti Immaturus*, *Citrus medica*, *Cyperus Rotundus,Wuyao*, *Fructus Toosendan*, *Caulis Perillae*, *Massa Medicata Fermentata*, *Fructus Hordei Germinatus*, *Fructus Crataegi*, *Semen Raphani*, *Herba seu Radix Cirsii Japonici*, *Herba Cephalanoploris*, *Radix Sanguisorbae*, *Sophora Japonica*, *Thuja orientalis*, *Herba*, *Agrimoniae*, *Radizoma Bletillae*, *Pollen Typhae*, *Radix Notoginseng*, *Radix Sangyisorbae*, *Rhizoma Chuanxiong*, *Olibanum*, *Myrrha*, *Rhizoma Corydelis*, *Radix Curcumae*, *Rhizoma Curcumae*, *Rhizoma Sparganii*, *Radix Salviae Miltiorrhizae*, *Herba Leonuri*, *Semen Persicae*, *Flos CArthami*, *Faeces Trogopterori*, *Achyranthes Bidentata Blume*, *Cyathula Officinalis Kuan*, *Squama Manitia*, *Lignum Dalbergiae Odoriferae*, *Fructus Liquidambaris*, *processed Rhizoma Pinelliae*, *Rhizoma Pinelliae*, *Rhizoma Arisaematis*, *Rhizoma Typhonii*, *Radix Aconiti Coreani*, *Radix Platycodi*, *Flos Inulae*, *Bulbus Fritillariae Thunbergii*, *Rhizoma Cynanchi Stauntonii*, *Fructus Trichosanthis*, *Bulbus Fritillariae Cirrhosae*, *Caulis Bambusae in Taeniam*, *Bitter Apricot Kernel*, *Sargassum Fusiforme*, *Radix Stemonae*, *Radix Stemonae*, *Loquat leaf*, *Radix Scutellariae*, *Sangbaipi*, *Semen Lepidii*, *Stir-baked Flos Farfarae*, *Radix Peucedani*, *Ferrosoferric Oxide*, *Os Draconis Fossilia*, *Semen Ziziphi Spinosae*, *Semen Biotae*, *Radix Polygalae*, *Cortex Albiziae*, *Radix Codonopsis*, *Radix Pseudostellariae*, *Radix Astragali*, *Rhizoma Atractylodis Macrocephalae*, *Rhizoma Dioscoreae*, *Glycyrrhiza Uralensis*, *Radix Panacis Quinquefolii*, *Ziziphus Jujuba*, *Radix Morindae Officinalis*, *Herba Cistanches*, *Rhizoma Curculiginis*, *Herba Epimedii*, *Cortex Eucommiae*, *Radix Dipsaci*, *Rhizoma Cibotii*, *Rhizoma Drynariae*, *Fructus Psoraleae*, *Frucyus Alpiniae Oxyphyllae*, *Cuscuia Japonica*, *Herba Cynomorii*, *Radix Angelicae Sinensis*, *Rehmannia glutinosa*, *Radix Polygoni Multiflori*, *Radix Paeoniae Alba*, *Radix Ophiopogonis*, *Herba Dendrobii*, *Bulbus lilii*, *Fructus Lycii*, *Herba Ecliptae*, *Ligustrum Lucidum*, *Radix Glehniae*, *Rhizoma Atractylodis Macrocephalae*, *Fructus Schisandrae*, *Fructus Tritici Levis*, *Radix Oryzae Glutinosae*, *Radix Ephedrae*, *Fructus Corni*, *Fructus Rosae Laevigatae*, *Fructus Rubi*, *Concha Arcae*, *Folium Ginseng*, *Radix Adenophorae.*
